# Insulin Resistance, Type 1 and Type 2 Diabetes, and Related Complications: Current Status and Future Perspective

**DOI:** 10.1155/2014/276475

**Published:** 2014-03-18

**Authors:** Joseph Fomusi Ndisang, Sharad Rastogi, Alfredo Vannacci

**Affiliations:** ^1^Department of Physiology, College of Medicine, University of Saskatchewan, 107 Wiggins Road, Saskatoon, SK, Canada S7N 5E5; ^2^Division of Cardiology, Department of Medicine, Henry Ford Heart and Vascular Institute, 2799 West Grand Boulevard, Detroit, MI 48202-2689, USA; ^3^Department of Pharmacology, Center for Integrative Medicine, Center for Molecular Medicine (CIMMBA), University of Florence, Viale Pieraccini 6, 50139 Florence, Italy

The global escalation of obesity and diabetes in developed and developing nations poses a great health challenge. Obesity is one of the major causes of type 2 diabetes [1, 2]. Type 1 diabetes is primarily due to the autoimmune-mediated destruction of pancreatic beta-cell leading to insulin deficiency [3, 4]. This is usually accompanied by alterations in lipid metabolism, enhanced hyperglycemia-mediated oxidative stress, endothelial cell dysfunction, and apoptosis [3, 4]. Similarly, in type 2 diabetes, increased glucotoxicity, lipotoxicity, endoplasmic reticulum-induced stress, and apoptosis lead to the progressive loss of beta-cells [3, 4]. While type 1 diabetes is characterized by the presence of beta-cell autoantibodies, a combination of peripheral insulin resistance and dysfunctional insulin secretion by pancreatic beta-cells is implicated in the pathogenesis of type 2 diabetes [3, 4]. Although insulin resistance has traditionally been associated with type 2 diabetes, mounting evidence indicates that the incidence of insulin resistance in type 1 diabetes is increasing [5, 6]; therefore novel mechanistic approaches deciphering insulin resistance are needed. Many pathophysiological factors are implicated in insulin resistance. Although the exact nature of these factors is not completely understood, it is becoming increasingly clear that oxidative stress, inflammation, genetic, habitual, environmental, and epigenetic factors may be involved [7–14].

In the past decade, significant strides have been made in elucidating important mechanisms associated with insulin resistance, overt diabetes, and related cardiometabolic diseases. However, more intense research is still needed for more comprehensive understanding of the pathophysiological profile of insulin resistance in diabetes, and especially in situations where diabetes is comorbid with other chronic diseases.

In this special issue research and review papers that address a broad range of mechanisms associated with insulin resistance, type 1 diabetes, type 2 diabetes, and related cardiometabolic complications are discussed. Accordingly, in a review article by X. Jiang et al. the impact of habitual and environmental factors on the development of diabetes is discussed. Particularly, X. Jiang et al. showed that nutritional, environmental, and physiological factors at prenatal age are correlated to the manifestation of insulin resistance and type 2 diabetes in later stages of adult life. On the other hand, healthy habits including exercise may prevent diabetes. Accordingly, C.-H. Hunget al. wrote an article showing the benefits of exercise on attenuating inflammation in diabetes and related complications. To further elucidate the pathophysiological role of inflammation on diabetes, Y. I. Sánchez-Zamora and M. Rodriguez-Sosa wrote a review article on the effects of macrophage migration inhibitory factor in type 1 and type 2 diabetes. In another related article, A. Zhu et al. reviewed the effects of abdominal adiposity on diabetes, while X. Zhang et al. reported the effects of fluctuating glucose levels on carotid artery intima-media thickness and the development of coronary artery disease. Besides vascular complications, many diabetic patients have increased risk of thrombosis due to insulin resistance and elevated inflammation [15]. This notion was further investigated in an article featuring in this special issue written by I. Isordia-Salas et al.

Among the major microvascular complications in diabetes are diabetic retinopathy and diabetic nephropathy. To shed more lights on these diabetic complications, X. Li et al. investigated the effects of oxidative stress on retinal neuron apoptosis, while G. Wu et al. reported the renoprotective effects of benidipine in streptozotocin-induced diabetic rats.

Taken together, the articles featuring in this special issue cover a broad range of themes of considerable interest and would benefit a wide audience.

*Joseph Fomusi Ndisang* *Joseph Fomusi Ndisang* *Sharad Rastogi* *Sharad Rastogi* *Alfredo Vannacci* *Alfredo Vannacci*

## References

[1] Hossain P, Kawar B, El Nahas M (2007). Obesity and diabetes in the developing world—a growing challenge. *The New England Journal of Medicine*.

[2] WHO

[3] Mishra M, Ndisang JF (2013). A critical and comprehensive insight on heme oxygenase and related products including carbon monoxide, bilirubin, biliverdin and ferritin in type-1 and type-2 diabetes. *Current Pharmaceutical Design*.

[4] Tiwari S, Ndisang JF (2013). The heme oxygenase system and type-1 diabetes. *Current Pharmaceutical Design*.

[5] Cleland SJ, Fisher BM, Colhoun HM, Sattar N, Petrie JR (2013). Insulin resistance in type 1 diabetes: what is “double diabetes” and what are the risks?. *Diabetologia*.

[6] Nokoff NJ, Rewers M, Cree Green M (2012). The interplay of autoimmunity and insulin resistance in type 1 diabetes. *Discovery Medicine*.

[7] Ndisang JF, Jadhav A (2010). The heme oxygenase system attenuates pancreatic lesions and improves insulin sensitivity and glucose metabolism in deoxycorticosterone acetate hypertension. *American Journal of Physiology: Regulatory Integrative and Comparative Physiology*.

[8] Ndisang JF, Lane N, Jadhav A (2009). Upregulation of the heme oxygenase system ameliorates postprandial and fasting hyperglycemia in type 2 diabetes. *American Journal of Physiology: Endocrinology and Metabolism*.

[9] Settin A, El-Baz R, Ismaeel A, Tolba W, Allah WA (2014). Association of ACE and MTHFR genetic polymorphisms with type 2 diabetes mellitus: susceptibility and complications. *Journal of the Renin-Angiotensin-Aldosterone System*.

[10] Schwenk RW, Vogel H, Schurmann A (2013). Genetic and epigenetic control of metabolic health. *Molecular Metabolism*.

[11] Miao F, Chen Z, Genuth S (2014). Evaluating the role of epigenetic histone modifications in the metabolic memory of type 1 diabetes. *Diabetes*.

[12] O'Keefe  JH, Bhatti SK, Patil HR, DiNicolantonio JJ, Lucan SC, Lavie CJ (2013). Effects of habitual coffee consumption on cardiometabolic disease, cardiovascular health, and all-cause mortality. *Journal of the American College of Cardiology*.

[13] Sandovici I, Hammerle CM, Ozanne SE, Constancia M (2013). Developmental and environmental epigenetic programming of the endocrine pancreas: consequences for type 2 diabetes. *Cellular and Molecular Life Sciences*.

[14] Murea M, Ma L, Freedman BI (2012). Genetic and environmental factors associated with type 2 diabetes and diabetic vascular complications. *The Review of Diabetic Studies*.

[15] Hess K, Grant PJ (2011). Inflammation and thrombosis in diabetes. *Thrombosis and Haemostasis*.

